# A Practical and Theoretical Treatise on the Diagnosis, Pathology, and Treatment of Diseases of the Skin, &c.

**Published:** 1843-07-01

**Authors:** 


A Practical and Theoretical Treatise on the Diagnosis, Pathology, and Treatment of Diseases of the Skin, arranged according to a Natural System of Classification, and preceded by an Outline of the Anatomy and Physiology of the Skin. By *Erasmus Wilson,* Consulting Surgeon to the St. Pancras Infirmary, Lecturer on Anatomy and Physiology, &c. London: Churchill, 1842. 8vo. pp. 407.

The pathology of the Skin is a subject of so much interest and importance that we are not sorry to meet with a work dedicated especially to its consideration. Since the time of Willan and Bateman, the subject has been much neglected in England, the field has been occupied by translations, and it is therefore refreshing to find British energy again bending to the task and seeking to maintain those rights that a long line of distinguished authors, from Daniel Turner downwards, have established. The author of the present volume is known to fame by his works on anatomy, and as the first chapter evinces, this subject and its twin sister Physiology have been laid deeply under contribution in order to plan out a fair foundation, on which as fair a superstructure, should, it might be hoped, arise. And truly, after a careful perusal of the work, we think that Mr. Wilson has succeeded in effecting the object he appears to have had in view, and we shall endeavour to give proof of our opinion by placing before our readers such quotations and comments as may be interesting either from their novelty or practical utility.

To the subject of Classification the author has devoted considerable attention, and he proposes and follows in the present volume a new system of arrangement, for which he claims the designation of the "*Natural System.*" But we quote his words:—

"The basis of the natural system of classification rests upon anatomy and physiology, and herein lies its strength, its simplicity, its easy application, and its truth. The dermis and its dependencies, its glands and its follicles, are the undoubted seat of all the changes which characterise cutaneous pathology. These, then, constitute my four primary divisions, namely—

Diseases of the Dermis,
Diseases of the Sudoriparous Glands,
Diseases of the Sebaceous Glands,
Diseases of the Hair and Hair-follicles."

He next enquires into the nature of the general diseases of these separate tissues, and lastly into their particular forms and the varieties of those forms. The author's arrangement is shown in the following table:—

### I. DISEASES OF THE DERMIS.

- Inflammation

  - Congestive

    - Specific: Rubeola. Scarlatina. Variola. Varicella. Vaccinia.

    - Non-specific: Erysipelas. Urticaria. Roseola. Erythema.

  - Effusive

    - Asthenic: Pemphigus. Rupia.

    - Sthenic: Herpes. Eczema. Sudamina.

  - Suppurative: Impetigo. Ecthyma.

  - Depositive: Strophulus. Lichen. Prurigo.

  - Squamous: Lepra. Psoriasis. Pityriasis.

  - From Parasitic Animalcules: Scabies.

- Hypertrophy of the Papillæ: Ichthyosis. Tylosis. Clavus. Verrucæ. Cornua.

- Disorders of the Vascular Tissue: Vascular Nævi. Purpura.

- Disordered Sensibility: Hyperesthesia. Pruritus.

- Disordered Chromatogenous Function

  - Augmentation of Pigment: Nigrities. Pigmentary Nævi.

  - Diminution of Pigment: Albinismus. Vitiligo.

  - Alteration of Pigment: Ephelis. Lentigo. Chloasma. Melasma.

  - Chemical Coloration: Oxyde of Silver Stain.

### II. DISEASES OF THE SUDORIPAROUS GLANDS.

| | |
|---|---|
| Augmentation of Secretion | Sudatoria. |
| Diminution of Secretion | |
| Alteration of Secretion | Abnormal Odour, Colour, &c. |

### III. DISEASES OF THE SEBACEOUS GLANDS.

| | |
|---|---|
| Augmentation of Secretion | Stearrhœa. |

| | | |
|---|---|---|
| Diminution of Secretion. | | |
| Alteration of Secretion | | Ichthyosis Sebacea. |
| Retention of Secretion | Duct Open | Comedones.<br>Sebaceous Accumulations.<br>Small Sebaceous Tumours, *(Molluscum Contagiosum.)* |
| | Duct Closed | Sebaceous Miliary Tubercles.<br>Calcareous Miliary Tubercles.<br>Serous Cysts.<br>Encysted Tumours. |
| Inflammation of Glands and adjacent Textures | | Acne.<br>Sycosis. |

IV. Diseases of the Hairs and Hair-Follicles.

| | |
|---|---|
| Augmented Formation | Pilous Nævi. |
| Diminished Formation | Alopecia.<br>Calvities. |
| Alteration of Colour | Canities. |
| Disease of the Hair-Pulps | Plica Polonica. |
| Disease of the Follicles | Inflammatio Folliculorum.<br>Favus. |
| Abnormal Direction | Trichiasis.<br>Felting. |

We consider this classification to be a decided improvement on that of Willan, which has been generally followed to the present day. The Willanean system is founded on the more obvious external characters of the disease, and is neither scientific or philosophical. For instance, if the disorder consist of vesicles, the disease belongs to the order *Vesiculæ;* if pustules, *Pustulæ;* if pimples, *Papulæ*; if scales, *Squamæ*, &c. Here then it would seem at first sight that a fixed and unsliding scale is laid down, by which the school-boy might jump to a correct diagnosis of the disease. But is this supposition founded in fact? We think not, nay, we know that the diagnosis of diseases of the skin is in the highest degree perplexing to many practitioners. But this is not all, the system involves other and greater objections; the same disease may and actually does, as in the case of small-pox, pass through several different orders of the Willanean Classification in its course. At first it is an *exanthem;* seen a little later it would be classed under *papulæ;* still later it is a *vesicular* affection, and after that it becomes a member of the order *pustulæ*, where we find it located by Willan. But we pass over this and other objections to the artificial system of Willan to enquire how far similar objections may be made to the classification of our author. It must be confessed that these do not exist: Mr. Wilson has based his system on the immutable principles of sound pathology, and he has, to use his own words, " created a system which should embrace all the advantages of the natural system (as applied to botany), while it retained all the benefits derivable from the artificial system." The ordinary principles of diagnosis employed in the Willanean system are equally applicable in the natural system; if the disease is vesicular, the practitioner will find it under the head of " Inflammation of the Skin terminating in Effusion;" if pustular, under Suppurative Inflammation; if scaly, under "Inflammation terminating in the Production of Squamæ," in other words, squamous inflammation; if papular, under inflammation terminating in deposition in the dermic tissues, &c. But the whole of these diseases are collected together under a designation indicative of their pathological state, namely, under that of "Inflammation of the Dermis." We need scarcely point out the therapeutic advantages of this arrangement, the general principles of treatment of inflammation must be applicable to the whole of the diseases included under that group, and these are nearly the whole of the affections commonly known as the diseases of the skin. Another feature in this classification is deserving of note: it is the retention of all the names hitherto used in reference to these diseases. "I should have esteemed," writes the author, "a natural and perfect classification as dearly purchased, at the sacrifice of a familiar nomenclature. I have scarcely changed a single term of Willan's glossary, and in the few instances in which I have departed from this rule, I have been guided by weightier considerations than those of accommodating diseases to a system of my own."

We have always regarded the extreme subdivision of diseases as one of the errors of cutaneous pathologists, and we are glad to find our author expressing the same sentiments. He observes, in his Preface, page 10,

"Another objection to the Willanean Classification is less important, but still a blemish in his system. I allude to the imitation of the divisions and subdivisions employed in the arrangement of zoological or botanical subjects. Thus, starting with a *Class*, Cutaneous Disorders, Willan established eight *Orders;* each of these Orders has its *Genera*, and Genera their *Species*. But pathological appearances do not admit of this gradation of subdivision, and no advantage can possibly flow from its adoption. The most that can be admitted is a class of Cutaneous Diseases, these divisible into orders, or groups; but the groups separate at once, to the exclusion of genera, into individual diseases, or species, and varieties of those diseases. The differences between any two varieties are never so strongly marked as to admit of consideration as species, in the proper sense of employing that term."

But leaving the subject of classification, which we have already permitted to engage so large a share of our attention, we will proceed to the other topics treated of in the volume. The first chapter, as we before observed, is devoted to the Anatomy and Physiology of the Skin, a knowledge of which, it is presumed, is absolutely necessary to the perfect comprehension of the pathology of the organ. We select from this chapter, as illustrative of the manner in which the author treats his subject, his description of the hair, with the manner of its growth, and development.

"Hairs are horny appendages of the skin, produced by the involution and subsequent evolution of the epidermis; the involution constituting the follicle in which the hair in enclosed, and the evolution, the shaft of the hair. Hairs vary much in size and length in different parts of the body; in some, they are so short as not to appear beyond the follicle, in others, they grow to an enormous length, as on the scalp; and along the borders of the eyelids, and on the beard, they attain to a very considerable thickness. Hairs are generally more or less flattened in their form, and when the extremity of a transverse section is examined with the microscope, it is found to present an elliptical or reniform outline. This view of a hair exhibits also an important fact with regard to its structure—namely, that the hair is porous and loose in texture in the centre, and dense in its circumference, affording some ground for the statement of its constitution of a cortical and medullary portion. The free extremity of a hair is generally pointed, and sometimes split into two or three filaments. Its attached extremity is implanted deeply in the integument, extending through the dermis into the subcutaneous areolar tissue, where it is surrounded by adipose vesicles. The central extremity of a hair is larger than its shaft, and is called the root, or bulb. It is usually infundibular in form in the larger hairs, and conical in the smaller hairs, and in those of the head.

"At the bottom of each follicle is a vascular and sensitive formative substance, or pulp, which is analogous to a papilla of the dermis, and is the producing organ of the hair. The process of formation of a hair by its pulp is identical with that of the formation of the epidermis by the papillary layer of the dermis. A stratum of plastic lymph is, in the first instance, exuded by the capillary plexus of the pulp; this plastic lymph, or blastema, undergoes conversion, firstly, into cyto-blasts, and then into cells; and these latter are lengthened out so as to correspond with the axis of the hair, and constitute a fibrous structure.

"This is the mode of formation of the greater part of the diameter of the hair; but the cells of the superficies comport themselves differently, in order to provide the polished surface which is characteristic of these structures. In this situation, as upon the surface of the epidermis, the cells are converted into flat scales, which enclose the fibrous structure of the interior. These scales, as they are successively produced, overlap those which precede and give rise to the rough and waving lines which may be seen around the circumference of a hair. It is this overlapping line that is the cause of the roughness which we experience in drawing a hair from its point to its bulb between the fingers, and the loosened state of the borders of these scales has given rise to the notion entertained by Leeuwenhoeck, of branches growing out from the shaft. The bulb is the newly-formed portion of the hair, it corresponds in figure with that of the pulp, and its expanded form is due to the greater bulk of the fresh cells, as compared with the fibres and scales into which they are subsequently converted in the shaft of the hair.

"The colour of hair, like that of the epidermis, is due to the presence of pigmentary granules, contained within the cells. In the white hair of Albinoes, there is a total absence of the colouring principle of these granules, and in some forms of the blanched hair of age, a white pigment supplies the place of the tint of early life." 11.

The second chapter treats of Diseases which are characterised by Congestive Inflammation of the Skin. Of the seat of congestive inflammation the author remarks:—

"The immediate seat of the inflammatory congestion of the exanthemata is the vasucular rete of the dermis, and the difference of tint observable in these diseases at their height and during their decline, is sufficiently explained by reference to the structure and normal phenomena of the skin. When the degree of excitation of the cutaneous nerves is small, and the arterial determination but little exalted above the ordinary standard, the vascular rete of the dermis is only partially congested, and the redness produced by this congestion is slight; such is the redness, with slight modifications depending on degrees of intensity of nervous excitement, which is seen in erysipelas, roseola, and erythema. When, however, the nervous activity is aroused to its highest pitch of energy, as in scarlatina, the congestion is most intense, and the bright scarlet of the arterial blood coursing through its vessels is little obscured by the thin veil of epidermis which binds it in its sphere. The congestion in rubeola, scarlatina, and variola, is not confined to the parallel strata of the vascular rete of the dermis, as in the second group of exanthemata, but many of the papillæ of the dermis are also distended with blood, and give rise to that punctiform appearance of the redness which is characteristic of these eruptions.

The crescentic form of the congested patches seen in rubeola, depends upon some unexplained peculiarity in the distribution of the cutaneous nerves, and corresponds with that natural appearance of the skin which is so frequently seen in healthy children, and which is denominated, mottled. Again, I have observed, that in injecting the limb of an infant with size and vermillion, I can imitate all the forms of redness seen in the exanthematous diseases, by ceasing to inject from time to time, or by filling the capillaries to their uttermost." 23.

In reference to the transmission of cutaneous disorders by contagion and infection, Mr. Wilson gives the following definition of those terms.

"In their more usual acceptation, the terms infection and contagion relate to modes of transmission of a poisonous principle. When the transmission is effected by a material substance, and is brought about by actual contact, the term *contagion* (immediate contagion) is employed; but when transmission is effected through the agency of the winds, and at a distance, the mode of communication is designated *infection,* (mediate contagion.) In other words, when the poisonous principle is volatile, and capable of diffusion in the atmosphere, it is infectious; but when this diffusibility is absent, it is simply contagious.

"In whatever way the poisonous principle be brought to the body of a sound person, and with whatever part of his body it may come in contact, whether with the cutaneous surface with or without abrasion, as in contagion, or with both the cutaneous and mucous surface in infection, the mode of its reception by the system is the same. In the first instance, it is dissolved in the fluids of the body, and, in the second place, is conveyed by imbibition into the circulating current of the blood, thence to act on the nervous system, and alter its functions. Once introduced into the system, the poisonous principle possesses the remarkable power of exciting an action similar to that which existed in the body whence it emanated, the intention of that action being the reproduction of an identical poison. Liebig has compared this process to fermentation; as, when a particle of yeast is brought in contact with a fermentable fluid, the particle of yeast is itself lost, or is too insignificant to be traced further; but the action which it excites occasions the formation of an abundance of similar yeast." 26.

In the treatment of Scarlatina, the author speaks favourably of cold affusion, to which he attributes a sedative effect on the nervous system. In the treatment of Variola, he compares the value of different *ectrotic-methods* which have from time to time been recommended, and bestows a merited encomium on the mercurial plaster of Vigo. The following observations on the influence of light in the development of the variolous pock are interesting.

"An impression subsisted among the ancient physicians, that the light of the apartment in which small-pox patients are kept, should be either modified or excluded. In pursuance of this view, the rooms were hung with scarlet cloth, and the windows carefully blocked up. So recently as 1832, Dr. Picton, of New Orleans, asserts, that in his practice no instance of pitting after small-pox occurred when the light was shut out. M. Serres placed a glass capsule over a small-pox pustule, and observed the effects produced by excluding the air and light. He found, that in proportion to the exclusion of both was the development of the pustule checked, and that when they were completely shut out, the pustule became shrivelled and quickly dried up. Moreover, M. Serres remarks, that he never reaped such successful results, in the cure of small-pox, as he did at La Pitié, during one year that the patients were placed in a kind of cellar, which was very dark and ill-ventilated. The same principle has been more recently acted on by M. Legrand, who proposed to the Academy of Medicine, in 1839, the plan of covering the surface of the body with gold leaf. After the experiments of M. Fourcault this practice would appear somewhat hazardous." 66.

"The author maintains that Rubeola, Scarlatina, and Variola, orginate in the same morbid contagion, the differences between them depending on modification either of the physical or of the vital condition of the system by which the contagion is received." Varicella, again, he considers to be an arrest of development of small-pox, and the forms which it assumes as capable of being deduced from the observation of the natural course of small-pox. Thus,

"Varicella, in this point of view, may be regarded as an arrest of development of variola, and the forms which it is capable of assuming may consequently be deduced from the observation of the natural course of small-pox. Thus, if the variolous disorder were to expend itself in its first stages, we should have a varicella resembling the papular eruption of small-pox, in other words, a *papular varicella;* if the variolous disorder progress beyond this stage, we shall then have a *vesicular varicella;* and if it proceed still further, a *pustular varicella.* The latter, however, is capable of presenting some modifications; in one of these, the contents of the conical vesicles are simply transformed into a purulent fluid, without any alteration of their form; this constitutes the *conical pustular varicella:* in another, the purulent fluid distends the vesicle to so great an extent, that it presents a globular figure; this is the *globular pustular varicella;* while in a third, the pustules assume the characteristic features of those of variola, being flattened and umbilicated; this, which is the most advanced grade of varicella, is the *umbilicated pustular varicella.*" 68.

On the subject of Variola Vaccinia, Mr. Wilson enters as largely as its importance deserves, treating successively of the identity of vaccinia and variola; of variola vaccinia in the cow and man, casual and vaccinated; of the secondary eruptions of vaccinia; of the protective power of vaccination; of the tests of vaccination; and of the various modes of restoring the influence of vaccination by revaccination; variolation after vaccination; retro-vaccination; variola-vaccination and recurrence to the primary vaccine vesicle. Of the theory of vaccination, he thus speaks:—

"It is a principle, well established in pathological science, that the animal system, once subjected to the influence of any disease originating in specific contagion, is protected, to a greater or less extent, against subsequent incursions of that disorder. Thus we observe that the modification which the system undergoes in the reception of rubeola and scarlatina, is protective of the individual against that contagion for the rest of life. The same circumstance is remarked with regard to small-pox, and other contagious fevers. When this fact was contemplated by the medical philosopher, in association with the fearful ravages of that dreadful pestilence and scourge upon the human race, small-pox, such as it existed in former ages, the expedient suggested itself to his mind, that if the disease could be anticipated, if the disorder, in a mild form, could be communicated to man, life would be spared, and the system equally defended against the subsequent contagion of a more virulent and fatal disease. This design, happy in thought, and happy in application, gave birth to the practice of inoculation for small-pox. Inoculation for small-pox, however, was not free from objection; the disease thus engendered was always serious, often fatal, and frequently it became the source of a malignant contagion. In this state of demi-subjugation, small-pox was found by Jenner, when the well-known fact of the protective influence of cow-pox first engaged his attention, and aroused in his comprehensive mind the philanthropic thought that spread happiness and security where gloomy anticipations and uncertainty had previously existed. He had the talent to perceive in cow-pox, small-pox, in its mildest possible form; and he trusted, that the transmission of this to man would ensure the same results as inoculation with the virus of human small-pox. This trust was rewarded, by the complete success which attended the prosecution of his views." 81.

The fourth chapter is occupied by the consideration of diseases characterised by effusive inflammation of the dermis, a group containing five diseases, Pemphigus, Rupia, Herpes, Eczema, Sudamina, corresponding with the two orders Bullæ and Vesiculæ of Willan. The fifth chapter is devoted to diseases characterised by suppurative inflammation, including Impetigo and Ecthyma, corresponding with the order Pustulæ of Willan. The sixth chapter treats of diseases consisting in "depositive inflammation of the dermis, corresponding with the order Papulæ of Willan, and embracing three diseases, Strophulus, Lichen, and Prurigo. While the seventh chapter contains the description of the squamous diseases. In the treatment of the latter affections, arsenic has long been a favourite remedy, and this has lately given place to a triple compound of iodine, arsenic, and mercury, the liquor hydriodatis arsenici et hydrargyri, suggested by Mr. Donovan of Dublin. Of the physiological effects of arsenic, Mr. Wilson remarks—

"Arsenic, when it acts on the nervous system, performs the part of an alterative: but when its effects are directed upon the digestive system, it appears to me to act like cantharides upon the mucous membrane of the kidney—viz. by counter-irritation, by exciting inflammatory action in the interior, and thus determining from the surface."

"The liquor hydriodatis arsenici et hydrargyri is exhibited in doses of half a drachm three times a day for the adult. It is liable to give rise to headache and nausea, and sometimes to salivation, during its use, and on the occurrence of these symptoms, it must be suspended for two or three days. The best vehicle for its exhibition is tincture of ginger, and it may be employed with advantage as a local application."

Chapter eighth is entitled "Inflammation of the Dermis induced by Parasitic Animalcules inhabiting the Epidermis." This definition applies only to Scabies, of which the author gives the following account.

"Scabies is an affection of the skin, characterised by scaliness of the epidermis, by vesicles, and in severe cases by pustules; to which may be added accidental abrasions and scratches produced by the nails. It is accompanied by excessive itching, the itching being augmented by warmth and by the use of stimulating food and drinks.

"The above appearances are due to the presence of a minute animalcule, the acarus scabiei, which burrows beneath the epidermis, and excites irritation in the papillary surface of the dermis. The burrowing of this little creature gives rise to the *scaliness* (scabrities) and undermined state of the epidermis. The vesicles, which are few and scattered, bearing no proportion to the number of the acari, and little relation to their seat, present some differences in form and character, respective of their position. Thus in the thin epidermis of the lateral surfaces of the fingers they are distinctly conical and acuminated; on the wrists and other parts of the body they are frequently more or less rounded, and resemble the vesicles of eczema; while in the latter situations they are also variable in size. The vesicles differ in reference to their contents; in those of a conical form, the contained fluid is transparent and viscous; in the rounded vesicle the fluid is also transparent, but in some it is more or less opaque and puriform. The pustules are present only in severe cases, or in persons with an extremely sensitive skin; they are generally psydracious, and vary in size, from the small pustule of impetigo to the larger pustule of ecthyma.

"When one of the early vesicles of scabies is examined with attention, a minute spot or streak, may be observed upon some one point of its surface. This is the aperture originally made by the insect on its first entrance beneath the epidermis, and from this spot or streak a whitish line may be traced either in a straight or a curved direction into the neighbouring epidermis. The whitish line is the *cuniculus*, or burrow of the acarus; it necessarily varies in length, being sometimes as much as five or six lines in extent, and at its termination, under a slight elevation of the epidermis, the little inhabitant lies concealed. The acarus may easily be distinguished by the experienced eye as a small dark point at the end of the cuniculus, and if a thin capsule of epidermis be raised in this situation with the point of a needle, the little creature is brought into view. It should be needless to remark, that eyes must be properly selected for the manipulation, and a bright light carefully chosen.

"The spot or streak which is here described is not met with on all the vesicles, for the same animal may excite a series of these in its course; and a number may be developed in the vicinity of its habitation, while in the primitive vesicle alone—that formed by the entrance of the acarus—it is, that the trace of its entrance can be expected. The aperture, again, does not communicate with the interior of the vesicle; it is the too close neighbourhood of the little *grubber* that acts as the cause of formation of the vesicle; the vesicle is consequently a provision of nature to protect the dermis from the nearer approach of the *arator*, and the vesicle is formed with the judgment which usually marks nature's operations—namely, before a defensive provision would be too late. The acarus scabiei, therefore, is *never* situated within the vesicle or within the pustule, and there is no communication between the vesicle and the cuniculus." 239.

In comparing the present with the preceding groups of diseases, Mr. Wilson remarks—

"The preceding groups of diseases, whether they originate in a local or a general cause, depend upon some pathological condition of the nerves and vessels of the system, or of the part affected. As a consequence of this pathological condition, we may have inflammation of the dermis in the various forms hereinbefore discussed—namely, congestive, effusive, suppurative, or squamous. The present group differs from the rest in obeying a specific cause, which may be present without exciting any general or local disorder of the nervous or vascular system, the seat of the cause being the extra-nervous and extra-vascular epidermis. When, however, the cause has been present for a certain period, varying with its number and with the temperament of the individual, we find such local effects produced as would result from the presence of the most common irritant. In the first instance, there is simple excitation of the peripheral nerves, giving rise to pruritus; next, there may be congestion of the capillary vessels; thirdly, there may be effusion of transparent lymph beneath the epidermis, constituting vesicles; and lastly, there may be suppuration, and the formation of pustules; each of these stages following an ascending grade of irritation; the degree in which the irritation is evinced depending in a greater measure on the temperament of the individual than upon the quantity of the cause.

"Guided by the Willanean classification alone, we should be led, seeing the alterations above described, in their first stage, to refer the disease to that group which includes erythema; in its second degree of severity, we might follow the example of all the dermatologists of the present day, and regard it as a vesicular disease, while in the highest and less frequent form of aggravation we should place it, as did Willan, among the pustules. It is clear, from the differences of such distinguished men, that any attempt to deduce its true position in cutaneous nosology from the accidental appearances respective of degree of irritation that it may present, must not only fail, but lead to serious errors in diagnosis. I have seen cases of scabies in which there were no vesicles and no pustules, but, nevertheless, the acarus revelled there in undisturbed enjoyment. Where would be the reputation of the medical practitioner who took no steps in such cases to protect the families in which they existed against the transmission of so repulsive a disease?" 288.

In the ninth chapter those diseases are assembled which depend on hypertrophy of the papillæ of the dermis; a pathological condition that, determined to be present in Icthyosis, causes the removal of that disease, from the squamæ of Willan, to a much more natural group, including corns, warts, and horns. The tenth chapter comprehends, under the title of disorders of the vascular tissue of the dermis, Nævus and Purpura. The eleventh chapter includes disorders of the sensibility of the dermis, and the twelfth, disordered colouration of the skin.

Diseases of the sudoriparous glands form the subject of chapter thirteen, and diseases of the sebaceous glands of chapter fourteen. Under the name of Icthyosis Sebacea, the author describes a remarkable form of concretion on the surface of the skin, simulating icthyosis, and, no doubt, sometimes mistaken for that disease.

"In addition to simple increase in quantity, it occasionally happens that the secretion of the sebaceous glands is also altered in its quality; when this is the case, the secretion spreads upon the surface of the epidermis, and forms a thin layer, which dries and hardens, and breaks in the direction of the linear markings of the skin into small polygonal portions, corresponding in form with the areæ of the compartments, bounded by these cutaneous lines. The small polygonal divisions are increased in thickness by the accumulation of fresh sebaceous secretion, they become discoloured from exposure to dust and dirt, and they assume a brownish or greyish tint, approaching more or less to dirt colour. In the latter state, the small masses have the appearance of scales, (icthyosis sebacea,) closely adherent to the epidermis, hard and dense in texture, and presenting various degrees of thickness. This affection may occur upon any part of the body, but is most frequent on the face, particularly on the forehead and the nose, upon the abdomen, and upon the flexures of joints; indeed, upon all those regions in which the greatest number of sebaceous glands exist, and which are most protected from the friction of dress. The scales are sometimes cast from time to time, particularly during the summer season, and give place to others formed by successive concretion; at other times they remain adherent for months, and even for years." 292.

This disorder has recently been made the subject of a pamphlet by Dr.

Jacobovics of Pesth, in which that author ascribes to it the name of "tubercules bigarrés," and erroneously considers it a new variety of Molluscum. On Molluscum Contagiosum Mr. Wilson disserts largely and advances some new cases of this disorder. He positively denies the contagious property ascribed to the disease by Bateman, and examines the evidence of their nature and history given by Tilesius, Bateman, Dr. John Thomson, Dr. Carswell, Alibert, Biett, Cazenave and Schedel, Gibert, Dr. Jacobovics, Dr. Henderson and Dr. Paterson.

The diseases of the Hairs and their Follicles engage the thirteenth Chapter. Ringworm is considered under the name, Favus, assigned to it by Rayer, and the complexity which has hitherto attended this disease is well explained by the author.

"No term has been more abused in medical nomenclature than has the word *porrigo*, and the confusion which exists in relation to the precise disease intended to be conveyed by the appellation will not cease, until the term is discarded altogether. It is with this view that I have made no use of it in the present treatise, other than as synonym. The species of porrigo of Willan, applied by that author to the disease under consideration, are two—*porrigo lupinosa,* corresponding with our favus dispersus; and *porrigo scutulata,* the favus confertus. A very common appellation for favus in the words and works of many of the most eminent English practitioners of the present day, and, indeed, the most correct term, is *porrigo favosa.* Here I conceive Willan erred; for both that author and Bateman employ the designation as significant of a variety of impetigo—impetigo of the scalp. *Porrigo larvalis* is impetigo faciei; *porrigo furfurans* appears to be an eczema, or, probably, pityriasis; and *porrigo decalvans* an alopecia." 345.

Speaking of the supposed vegetable nature of this disease, Mr. Wilson observes, and very justly,

"However closely the fungous growth here may resemble a plant, its vegetable nature is very far from being established. The simplest forms of animals are composed, like the mycodermis, of cells, variously connected together; and subsequent research may prove the growth under consideration to be of a similar nature. To my mind there is nothing improbable in the supposition of the origin of the growth from morbidly developed epidermic cells of the hair-follicle, or from the corpuscules of the sebaceous substance. In a preceding section of this work, I have shewn that the latter are susceptible of considerable alteration, and that in this state they assume an appearance widely different from that of their normal condition. Mr. Busk also entertains doubts with regard to the vegetable nature of the mycodermis, and deduces an opinion favourable to his opinion, from the chemical analysis of the crusts of favus, given by Thenard, who found them composed of

| | |
|---|---|
| Albumen . . . | 70 |
| Gelatine . . . | 17 |
| Phosphate of lime . . | 5 |
| Water and loss . . | 8 |
| | 100." |

Syphilitic affections of the skin form the subject of chapter sixteen; while the seventeenth and last is occupied by the history and description of the entozoa of the skin, the acarus scabiei and folliculorum; the preparation and therapeutic effects of two new medicines, Anthrakokali and Fuligokali; and a notice of three cases of a very rare disease, the Sudatoria, which occurred in Paris during the summer of 1842.

The Acarus folliculorum was discovered by Dr. Simon of Berlin, and a paper describing his discovery with figures of the animal, appeared in Müller's Archive for June 1842. The description of the animal given by the discoverer is however very imperfect as regards its anatomical structure. Mr. Wilson, in the work before us, makes some additions to the anatomy of the animal, and in a paper recently read before the Royal Society finds reason to alter the name of the creature, to entozoon folliculorum. In this paper he gives an elaborate description of the anatomy of the Entozoon, shows that it differs essentially from the generic characters of Acarus, and discovers its ova and mode of development, the latter being an elaborate and curious process. Of his success in finding the animal after perusing the paper of Dr. Simon, he thus speaks,—

"I was not long in obtaining subjects: almost every face that I met supplied me with abundance, and the difficulty seems to be, not to find the creature, but to find any individual, with the exception, according to Dr. Simon, of newly-born children, in whom these animalcules do not exist. It is by no means necessary to commence our search by selecting an acne punctatum, or even a comedo; almost every collection of sebaceous substance which can be squeezed forth from the numberless cutaneous apertures upon the nose, the forehead, the face, and probably from other parts of the body, will furnish subjects. Moreover, Dr. Simon has observed that the parasites are situated near the mouth of the follicle, consequently, that portion of sebaceous substance which is squeezed out with the least force is the part which is most likely to be inhabited by the acarus.

"The acarus folliculorum would seem to give rise to no uncomfortable effects by its presence, unless, perchance, it should multiply to such an extent as to become a source of irritation to the follicle—a supposition which Dr. Simon admits, for it is found in persons whose skin is perfectly healthy and clear, and in whom no signs of cutaneous irritation are present. These animalcules undoubtedly feed on the sebaceous substance in which they lie embedded, and which is the cause of their existence. I have commonly found two in the small mass of this substance expressed by the fingers, often four and five, and in one instance, eight closely connected together. Hitherto I have confined my examinations to living persons, having levied for contributions among my more intimate friends, and have not as yet had recourse to a skin studded with acne." 389.

We have now reached the conclusion of the volume and our perusal has been both agreeable and instructive. The work is well got up, it is illustrated with a wood-engraving title-page of the entozoon folliculorum, and presents an excellent key to its matter in an ample table of contents, and alphabetical index. The book is not written for a day but for an age, the style is good and precise, the language well selected, and the information which it contains genuine and copious. We think it adapted to cast a new light on the pathology and treatment of diseases of the skin and to form an admirable guide to the medical practitioner, to whom and to the student we warmly recommend it.

